# Low-Grade Appendiceal Mucinous Neoplasm Mimicking Pelvic Inflammatory Disease: A Case Report

**DOI:** 10.7759/cureus.111559

**Published:** 2026-06-26

**Authors:** Samuel Hernández Alvarado, José Rodrigo Alcántara Dzib, Wilbert José Cámara Valencia, Rodrigo Villarreal Zavala, Pedro Daniel Román Chavéz

**Affiliations:** 1 Surgery, Universidad Autónoma de Yucatán, Merida, MEX; 2 Pathology, Hospital General Regional No. 1 Ignacio García Téllez, Mérida, MEX; 3 Surgery, Hospital General Regional No. 1 Ignacio García Téllez, Mérida, MEX

**Keywords:** conventional right hemicolectomy, low grade appendiceal mucinous neoplasm, pelvic inflammatory disease (pid), pseudomyxoma peritonei, pyometra uteri

## Abstract

Low-grade appendiceal mucinous neoplasms (LAMN) are rare clinical entities. These lesions are characterized by an expansive growth pattern and a significant propensity for peritoneal dissemination, potentially leading to pseudomyxoma peritonei. Preoperative diagnosis of appendiceal mucinous neoplasm remains a formidable challenge, particularly in female patients, as clinical presentations often mimic common gynecological pathologies. This report describes the case of a 63-year-old female with a history of schizophrenia and previous pelvic surgeries who presented with generalized colicky abdominal pain, vomiting, and diarrhea. Initial diagnostic workup, including computed tomography, suggested a massive pyo/hydrometra and severe pelvic inflammatory disease, for which she received intravenous antibiotic therapy. Despite treatment, persistent pain led to a follow-up imaging study revealing a significant pelvic collection. During exploratory laparotomy, free mucin was identified in the right iliac fossa, and intraoperative biopsy reported acellular mucinous implants. A right hemicolectomy with a terminal ileostomy was performed after identifying a perforated appendix with a tumor base exceeding two centimeters. Histopathological analysis confirmed a low-grade appendiceal mucinous neoplasm, staged as pTis N0 pM1a (Stage IVA), with 16 negative lymph nodes and clear surgical margins. This case illustrates the diagnostic complexity of LAMN in elderly women, where mucinous collections can accurately simulate intrauterine or pelvic inflammatory processes such as massive pyo/hydrometra. It highlights the necessity of including appendiceal neoplasms in the differential diagnosis of complex pelvic masses to avoid iatrogenic rupture and optimize surgical outcomes. Precise surgical technique and long-term oncological surveillance remain critical, as the risk of late recurrence and progression to pseudomyxoma peritonei persists even in cases with acellular mucin and negative margins.

## Introduction

Appendiceal neoplasms are uncommon clinical entities, accounting for approximately 0.5% of all gastrointestinal tumors [1]. Low-grade appendiceal mucinous neoplasms (LAMNs) are found in fewer than 1% of appendectomies performed [2]. LAMN is notable for its unique biological behavior, characterized by expansile growth without destructive infiltrative invasion, but with a marked propensity for rupture and peritoneal dissemination [3]. The clinical significance of these lesions lies in their potential progression to pseudomyxoma peritonei (PMP), defined by the accumulation of mucin and tumor cells within the abdominal cavity [4].

The preoperative diagnosis of LAMN represents a significant clinical challenge due to its asymptomatic or nonspecific presentation. In women, this challenge is even greater, as clinical manifestations and imaging findings often mimic common gynecologic conditions, such as pelvic inflammatory processes or intrauterine collections, leading to diagnostic errors or incidental intraoperative findings [2,5].

Despite advances in imaging techniques such as computed tomography (CT), distinguishing between an adnexal mass and an appendiceal mucinous neoplasm remains complex, particularly when the presence of extraluminal mucin simulates purulent or inflammatory collections [6]. Misdiagnosis may result in inappropriate surgical management, increasing the risk of iatrogenic appendiceal rupture and peritoneal seeding [7,8].

We present the case of a 63-year-old female patient whose clinical presentation and tomographic findings initially suggested severe pelvic inflammatory disease (PID). However, the surgical approach and histopathologic examination revealed a low-grade appendiceal mucinous neoplasm (LAMN). This report highlights the importance of including appendiceal neoplasms in the differential diagnosis of complex pelvic masses and uterine collections in older adult women in order to optimize the diagnostic and surgical approach.

## Case presentation

A 63-year-old female patient, G2, P1, L1, A1, with a history of angle-closure glaucoma, untreated schizophrenia, allergy to metamizole and risperidone, instrumented uterine curettage 43 years prior, and bilateral tubal occlusion 22 years prior. Her current illness began two months before his hospitalization, with vomiting and generalized colicky abdominal pain, accompanied by occasional vomiting and diarrheal stools. She was treated on an outpatient basis for acute diarrheal disease; however, she had an unfavorable clinical course. Therefore, she was admitted to the hospital.

Upon admission, her vital signs were within normal limits, with no relevant laboratory abnormalities (Table 1), except for anemia with a hemoglobin level of 9.7 g/dL. On physical examination, she presented with pain in the hypogastrium and right iliac fossa on moderate palpation. There were no palpable masses at this level; pelvic examination revealed cervical motion tenderness, and speculum examination showed mucopurulent cervical discharge. A vaginal discharge culture was also obtained, which was positive for Candida glabrata. An abdominopelvic CT scan was performed, showing an intrauterine collection with massive pyo/hydrometra (Figure 1).

**Table 1:** Relevant laboratories during hospitalization. WBC: White Blood Cell Count; Hb: Hemoglobin; Hct: Hematocrit; PLT: Platelets / Platelet Count; Gluc: Glucose; Cr: Creatinine; LDH: Lactate Dehydrogenase; CA-125: Cancer Antigen 125; CA 19-9: Cancer Antigen 19-9; CEA: Carcinoembryonic Antigen; AFP: Alpha-Fetoprotein; CRP: C-reactive protein

Test name	Admission labs	Pre-surgical labs	Post-surgical labs	Postoperative follow-up	Hospital discharge labs
WBC (10³/uL)	7.0	9.07	35.6	30.6	9.1
Neutrophils (%)	59	76	89	91	69
Hb (g/dL)	9.9	11.0	13.7	13.2	8.9
Hct (%)	29.6	32.3	40.2	38	27.0
PLT (10³/uL)	350.0	336.0	340.0	303.0	235.0
Gluc (mg/dL)	103	91	148	130	84
Cr (mg/dL)	0.6	0.5	0.5	0.8	0.5
Sodium (mmol/L)	139	139	128	140	135
Potassium (mmol/L)	4.0	3.4	3.5	3.5	3.6
LDH (IU/L)	132	Not analyzed	253	197	Not analyzed
Albumin (g/dL)	3.1	Not analyzed	2.5	2.7	Not analyzed
CA-125 (U/mL)	Not analyzed	Not analyzed	Not analyzed	22	Not analyzed
CA 19-9 (U/mL)	Not analyzed	Not analyzed	Not analyzed	3.1	Not analyzed
CEA (ng/mL)	Not analyzed	Not analyzed	Not analyzed	<0.31	Not analyzed
AFP (IU/mL)	Not analyzed	Not analyzed	Not analyzed	4.8	Not analyzed
CRP (mg/dL)	Not analyzed	2.4	0.6	6.1	2.05

**Figure 1:**
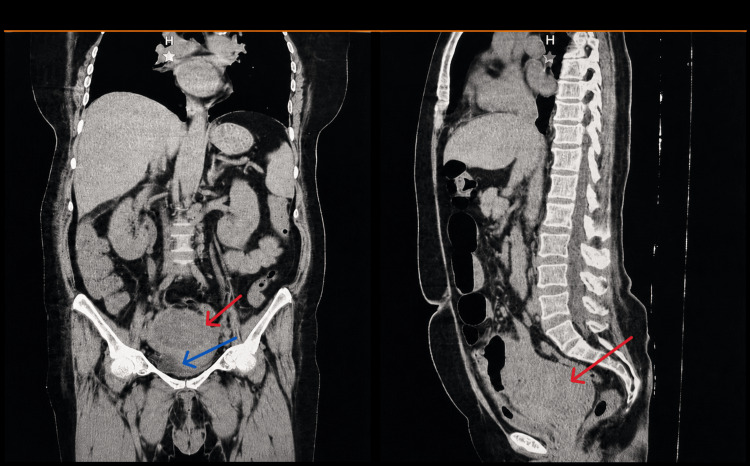
Abdominopelvic CT scan, coronal and sagittal sections. Distension of the uterine cavity is observed due to a collection with an air-fluid level measuring 108x91x111 mm, with endometrial thickening up to 78 mm (Red arrows), with displacement and compression of the bladder (Blue arrow).

In this context, she was treated as having severe pelvic inflammatory disease, completing seven days of dual antibiotic therapy with ceftriaxone/metronidazole.

During her clinical course, the patient continued to present abdominal pain predominantly in the lower quadrants; however, her leukocyte count remained within normal ranges. A follow-up contrast-enhanced abdominopelvic CT scan showed a 135-cc pelvic collection (Figure 2), for which she was scheduled for exploratory laparotomy. An exploratory laparotomy was performed with the following findings: free mucin at the level of the right iliac fossa. A sample was obtained for intraoperative examination, and the pathology report described two implants of acellular mucin.

**Figure 2:**
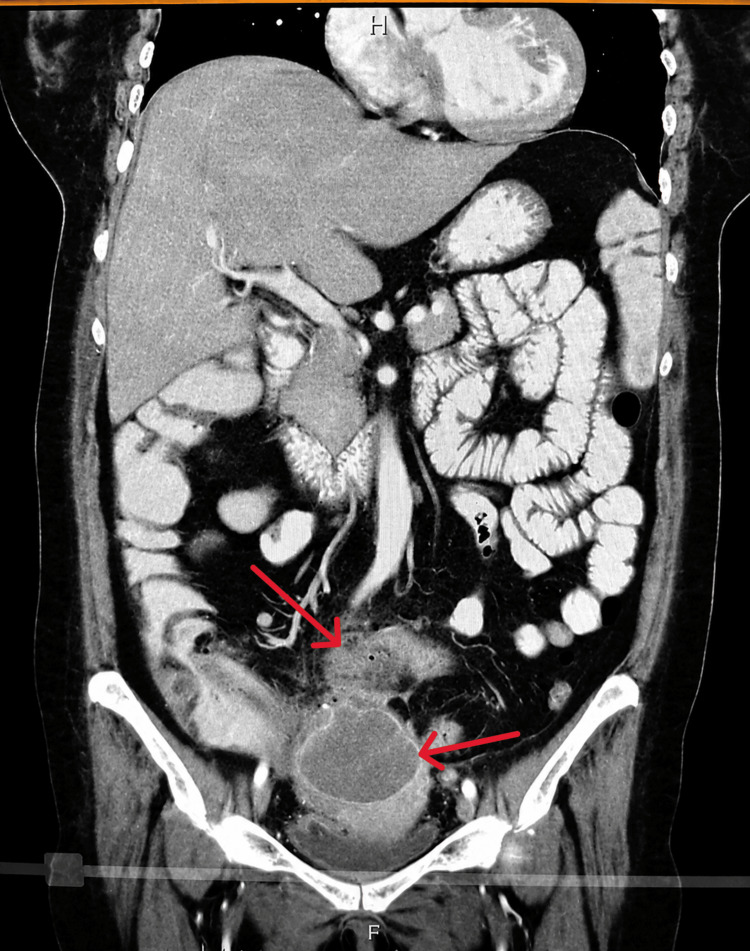
Contrast-enhanced abdominopelvic CT scan with oral contrast, coronal section. Pelvic collection in the hypogastrium, measuring 78x69x52 mm, with gas inside and a mass effect on the rectosigmoid, uterus and both ureters (Red arrows).

An intraoperative consultation with general surgery was requested because the institution did not have a surgical oncologist available. General surgery then continued the procedure, performing a right hemicolectomy, terminal ileostomy, and aspiration of mucin confined to the right lower quadrant. Intraoperative findings included bowel-to-abdominal wall and interloop adhesions, Zühlke grade III; a perforated, phlegmonous cecal appendix with mucinous fluid; and an appendiceal base with a tumor-like appearance measuring more than 2 cm (Figure 3). A hyperemic uterine fundus adhered to the appendiceal plastron was observed. In the right adnexa, the infundibulum and ampulla of the fallopian tube were abscessed and necrotic, with complete loss of the anatomy of the fimbriae and right ovary. Left adnexa demonstrated an atrophic ovary, with the remaining features being within normal limits.

**Figure 3:**
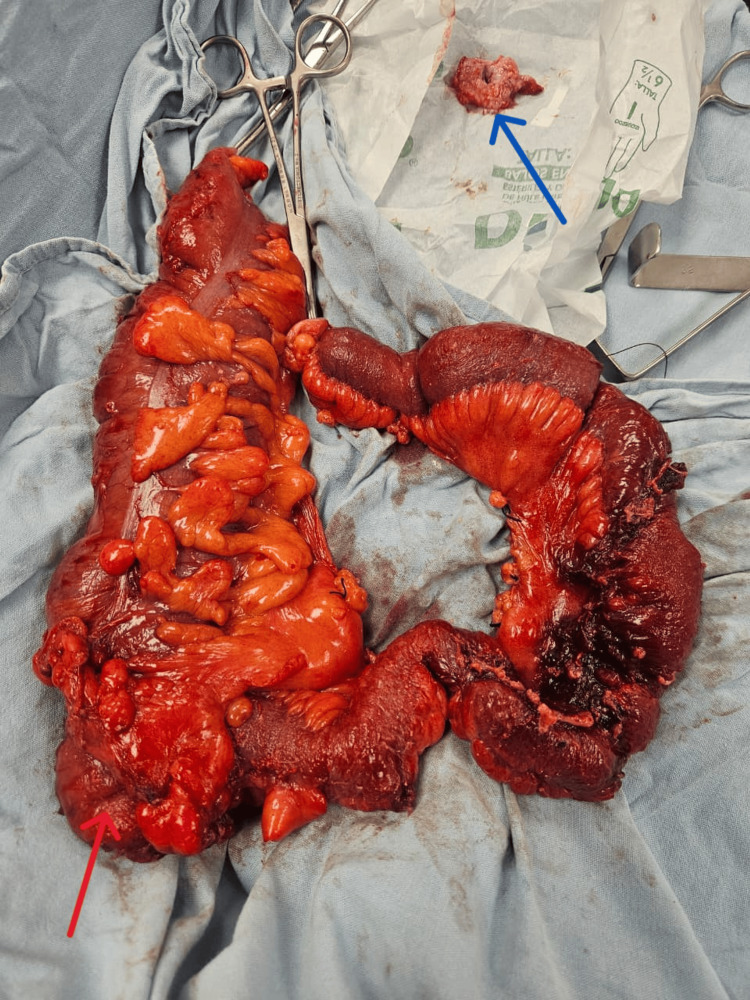
Surgical specimen from right hemicolectomy. A tumor-like appendix is observed (red arrow), as well as ileal hyperemia and serosal denudation secondary to adhesiolysis (due to firm, Zühlke grade III adhesions). Additionally, the right fallopian tube is observed, which was firmly adhered to the appendiceal plastron and underwent lysis (blue arrow).

The patient was admitted to the intensive care unit due to septic shock following the procedure. She showed favorable clinical progress after 48 hours and was discharged from the ICU, continuing surveillance in the hospital ward. Tumor markers were obtained and were within normal ranges (Table 1). Seven days after surgery, the patient progressed favorably, the ileostomy was functional, and she tolerated oral feeding; therefore, she was discharged due to clinical improvement.

The histopathologic report concluded (Figures 4, 5): Low-grade appendiceal mucinous neoplasm (LAMN), histologic grade G1; appendix with a 2.5 × 2 cm tumor extending into the lamina propria; 16 of 16 dissected lymph nodes negative for metastasis; all surgical margins negative for neoplasm. Surveillance will be continued by medical and surgical oncology for at least five years; the patient was scheduled for a six-month follow-up, at which time she would be evaluated with a CT scan.

**Figure 4:**
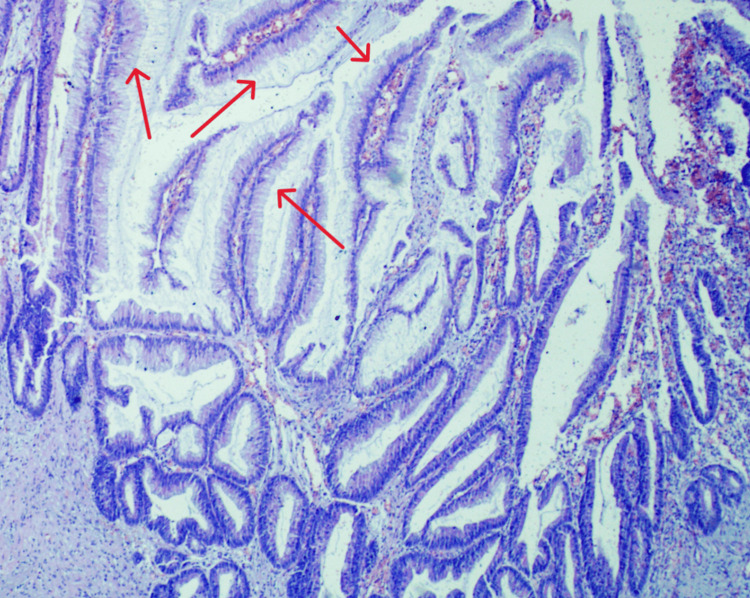
Appendix histopathological image. Photograph showing replacement of the appendix mucosa by a filiform villous mucinous epithelial proliferation (Red arrows). Hematoxylin and eosin stain. 10x.

**Figure 5:**
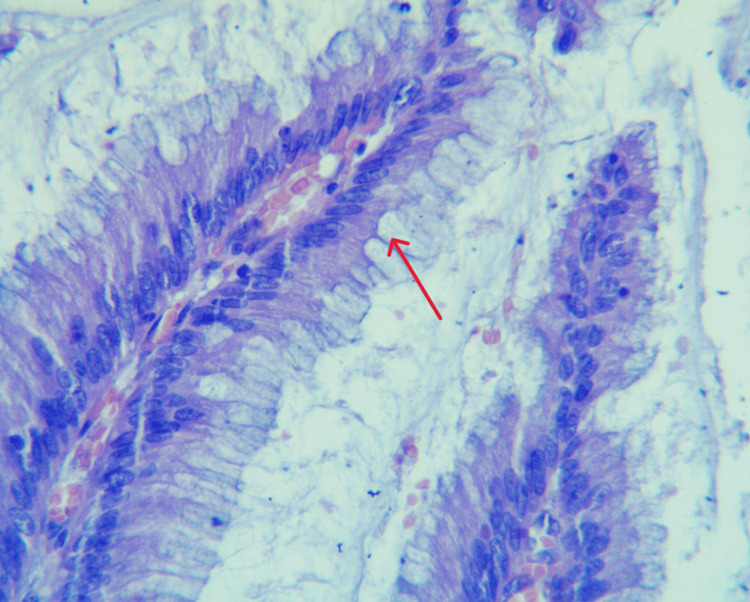
Low-grade mucinous neoplasm of the appendix (LAMN). Histopathological image. LAMN; characterized by columnar cells with cytoplasmic mucin vacuoles (Red arrow). Hematoxylin and eosin stain. 40x.

## Discussion

The present case illustrates the diagnostic complexity of LAMN in older adult women. The patient’s clinical presentation, characterized by lower abdominal pain and tomographic findings interpreted as massive pyo/hydrometra, initially led to a diagnosis of severe pelvic inflammatory disease (PID). This clinical mimicry is a documented phenomenon; LAMNs often present as adnexal tumors or pelvic inflammatory processes, delaying timely oncologic diagnosis until the surgical procedure in a large proportion of cases [2,5].

A distinctive aspect of this report is the simulation of pelvic inflammatory disease. In the literature, the presence of extraluminal mucin due to appendiceal rupture may be mistaken for purulent collections or tubo-ovarian abscesses on imaging studies [7]. As noted by Suárez-Velázquez et al., computed tomography is the imaging modality of choice, but its interpretation depends on clinical suspicion; findings such as appendiceal distension greater than 15 mm or the presence of intraluminal gas should raise suspicion for LAMN, even in clinical scenarios suggestive of PID [7,8].

From a pathologic perspective, the diagnosis of LAMN is based on the pattern of “pushing invasion” and the presence of mucinous epithelium with low-grade atypia [3]. In our patient, the histopathologic report confirmed stage pTis disease, confined to the lamina propria; N0, with 16 negative lymph nodes; and pM1a, defined as intraperitoneal acellular mucin without identifiable tumor cells in the disseminated peritoneal mucinous deposits. Tumor markers, including CEA, CA 19-9, and CA 125, were within normal ranges. This profile is consistent with the criteria of the AJCC 9th edition, which associates stage IVA disease with a 5-year survival rate of 63.24%, as reported in the study by Janczewski [9].

The surgical management of LAMN remains a subject of ongoing debate. Traditionally, right hemicolectomy (RHC) has been performed in cases with suspected malignancy or involvement of the appendiceal base. However, recent systematic reviews suggest that appendectomy with negative margins may be sufficient for non-disseminated LAMN, given that the incidence of lymph node metastasis is extremely low (<1%) [10]. In our case, the decision to perform RHC was justified by the intraoperative finding of a perforated appendix with a tumor-like base measuring more than 2 cm, as well as intraperitoneal acellular mucin. Nevertheless, the finding of 16 lymph nodes negative for malignancy and free surgical margins reinforces the theory that RHC does not always provide an additional oncologic survival benefit if complete resection of the primary tumor is achieved [1,10].

Finally, the risk of progression to pseudomyxoma peritonei (PMP) dictates the need for close follow-up. Although the patient had negative tumor markers and underwent successful resection, the presence of intraperitoneal acellular mucin increases the risk of long-term recurrence [4]. Surveillance with imaging studies and tumor markers every 6 to 12 months for at least 5 years is recommended, as PMP may develop years after initial treatment [4,11].

## Conclusions

Low-grade appendiceal mucinous neoplasm (LAMN) represents a diagnostic challenge in clinical practice. This case highlights that, in older adult women, complex pelvic or uterine collections - such as massive pyo/hydrometra - should not be attributed exclusively to gynecologic inflammatory processes. Although computed tomography is a valuable tool and is the study of choice over other imaging modalities, such as MRI, its interpretation may be equivocal when mucin mimics purulent collections, leading to misdiagnoses of pelvic inflammatory disease. The intraoperative finding of a dilated appendix or the presence of mucin should alert the surgeon to the need for a meticulous technique to avoid iatrogenic rupture and the consequent risk of pseudomyxoma peritonei.

Finally, although a right hemicolectomy was performed in this case due to suspected extensive oncologic involvement, current evidence suggests that appendectomy with negative margins may provide an excellent prognosis. Regardless of the surgical approach, close outpatient follow-up is mandatory to detect late recurrences.
